# Tag, you’re it!: viral diseases in native otters of south-central Chile due to coexistence with invasive American mink and domestic dogs

**DOI:** 10.3389/fvets.2025.1634282

**Published:** 2025-11-13

**Authors:** Alexis Santibañez, Cristina Coccia, Erwin M. Barría, Sandro Huenchuguala, Macarena Barros, Carlos Calvo-Mac, Gonzalo Medina-Vogel

**Affiliations:** 1 Programa de Doctorado en Conservación y Gestión de la Biodiversidad, Universidad Santo Tomás, Santiago, Chile; 2 Wenuleufu Center for Environmental Studies and Education, San Pablo, Chile; 3 Department of Science, University of Rome Tre, Rome, Italy; 4 National Biodiversity Future Center (NBFC), Università di Palermo, Palermo, Italy; 5 Bahia Lomas Research Centre, Santo Tomás University, Santiago, Chile; 6 Centro de Investigación e Innovación Sobre el Cambio Climático, Facultad de Ciencias (CiiCC), Universidad Santo Tomás, Santiago, Chile; 7 Department of Basic Sciences, Faculty of Sciences, Santo Tomás University, Puerto Montt, Chile; 8 Escuela de Tecnología Médica, Facultad de Salud, Universidad Santo Tomás, Los Carreras, Osorno, Chile; 9 Instituto One Health, Universidad Andrés Bello, Santiago, Chile; 10 PhD Program in Conservation Medicine, Facultad de Ciencias de la Vida, Universidad Andrés Bello, Santiago, Chile

**Keywords:** parvovirus, canine distemper virus, bridge host, metareservoir, serological detection, genetic screening

## Abstract

**Introduction:**

Biological invasions represent a significant epidemiological route for the introduction and dispersion of pathogens, facilitating disease emergence and transmission among native biodiversity. In the temperate rainforest ecoregion of south-central Chile, the native semiaquatic mustelid *Lontra felina* (marine otter) and *L. provocax* (southern river otter) coexist both sympatrically and syntopically with two invasive species—American mink (*Neogale vison*) and domestic dog (*Canis lupus familiaris*), that act as carriers and hosts of canine parvovirus and distemper.

**Methodology:**

To assess the occurrence of both diseases, we: (1) collected serum and mucous membrane samples from four species across three sectors of this ecoregion; and (2) employed serological immunoassays (IgG) and genetic analyses (qPCR-HRM) to detect both active and past infections, and to genotypically characterize the two viral agents.

**Results:**

75% of *L. felina* individuals tested positive for parvovirus. The melting temperature (T_m_) of the analyzed DNA fragment revealed two diverging groups, suggesting the presence of two genotypic variants of the virus within this mammalian assemblage. *L. felina* individuals carried the variant with the higher T_m_, which was also detected in *N. vison* from the same locality. In contrast, *L. provocax* individuals carried the variant with the lower T_m_, while dogs and minks hosted both viral variants. Canine distemper virus was detected only in dogs that also tested positive for parvovirus.

**Discussion:**

Our results present the first report of parvovirus in *L. felina* and support the hypothesis that *N. vison* and dogs acts as metareservoir and mink also as a bridge host for its transmission. In the study area, the synanthropic behavior of *N. vison* and its interactions with domestic and native species may facilitate the diversification of emergent pathogens within Chilean native fauna.

## Introduction

Biological invasions promote the biodiversity loss by intensifying predation and competition pressures, causing environmental disruption, and/or facilitating disease transmission (1–3). The diversification of exotic pathologies in native ecosystems is primarily facilitated by interactions between endemic fauna and invasive species, the latter serving as reservoirs for some diseases (4, 5). Therefore, assessing the prevalence of different diseases in native and invasive fauna not only enables the understanding the effects of species invasions, but also provides insight into their implications for human health, which is particularly relevant given that recent pandemics have arisen from interactions between humans and wildlife with zoonotic consequences (6–10).

The temperate rainforest of south-central Chile is an ecoregion situated toward the southern end of the Chile Central biodiversity hotspot (11, 12). Over the past three centuries, this biome has undergone significant environmental impacts, including habitat loss, fragmentation, forest fires, and reduction of biodiversity by introduction of invasive species driven by urbanization, industrialization, agricultural and forestry activities (13–15). Here the endangered southern river otter (*Lontra provocax*) is fund. It is a species notable for its extremely low density and dependence on pristine riparian habitats (16, 17). In addition, the congeneric and endangered marine otter (*L. felina*) is more frequently observed in relatively undisturbed rocky coastal systems (18, 19). Both species coexist with the American mink (*Neogale vison*), a highly dispersive exotic mustelid whose range in Chile has expanded 2,500 km northward from its center of origin in southern Patagonia over the past century (20–23). Further, the domestic dog (*Canis lupus familiaris*) is an invasive species intrinsically linked to human activities that combines high synanthropy due to domestication, with a tendency to expand their home range and form packs that prey on native species (24–30). Additionally, dogs are globally recognized as the primary reservoir of canine parvovirus and canine distemper viruses, serving as the principal source of origin and diversification of both pathogens in wildlife worldwide (31–35).

Both diseases have multi-host capacity facilitated by their ability to remain viable in the air for weeks and by their transmission routes, which include aerosolized secretions and passive spread via fomites (36–38). In south-central Chile, domestic dogs exhibit active movement dynamics between rural, semi-rural, and forested areas, with a canine parvovirus and canine distemper seroprevalences ranging between 50 and 70% (39, 40). Both diseases have also been detected in minks and southern river otters, suggesting that mink acts as an active transmission bridge, as they move through forested areas and interact with domestic dogs, particularly when they hunt in poultry farms. Furthermore, southern river otters are particularly vulnerable to infection by these pathogens, as various native and invasive mammal species co-occur at their latrines, where fecal and urine deposits serve as focal points of infection for this and other species attracted to these conspicuous places (41–43).

Immunologically, the infections with canine parvovirus and canine distemper trigger antigen–antibody binding reactions involving immunoglobulin G or IgG (44). This enables the use of specific serological immunoassays to detect past infections by identifying the presence of antibodies (45). Complementarily, the amplification of specific viral gene fragments enables the detection of the pathogenic agent currently present in the organism (46, 47). Quantitative real-time PCR (qPCR) combined with high-resolution melting analysis (HRM) enables the characterization of nucleotide sequences based on the temperature required to denature half of a double-stranded DNA strand, a property known as the melting temperature or T_m_ (48, 49). Thus, variations observed in T_m_ peak ranges indicate structural differences in gene sequences caused by specific mutations, which deviate from the known structure of a reference sequence (50, 51). Therefore, immunoassays are effective in detecting past infections; however, they lack the specificity required for detailed pathological characterization. Nevertheless, these techniques exhibit functional complementarity with genetic analyses based on DNA sequences (e.g., qPCR-HRM), which enable the detection of active infections as well as the genetic typing of the causative agents of parvovirus, particularly canine parvovirus (52–54).

We employed serological methods based on antigen–antibody reactions to test for parvovirus and distemper infections in dogs, as well as invasive mink, marine and southern river otters, across three distant locations within the temperate rainforest ecoregion of south-central Chile. Additionally, qPCR-HRM was performed to detect the presence of genetic material from these pathogens and to infer structural variability in the amplified fragment sequences based on their T_m_ peak ranges. These results will allow us to establish genetic-structural associations between viral agents affecting native fauna and to infer the role of invasive fauna as vectors of pathogen transmission.

## Materials and methods

### Sample collection

**Figure 1:**
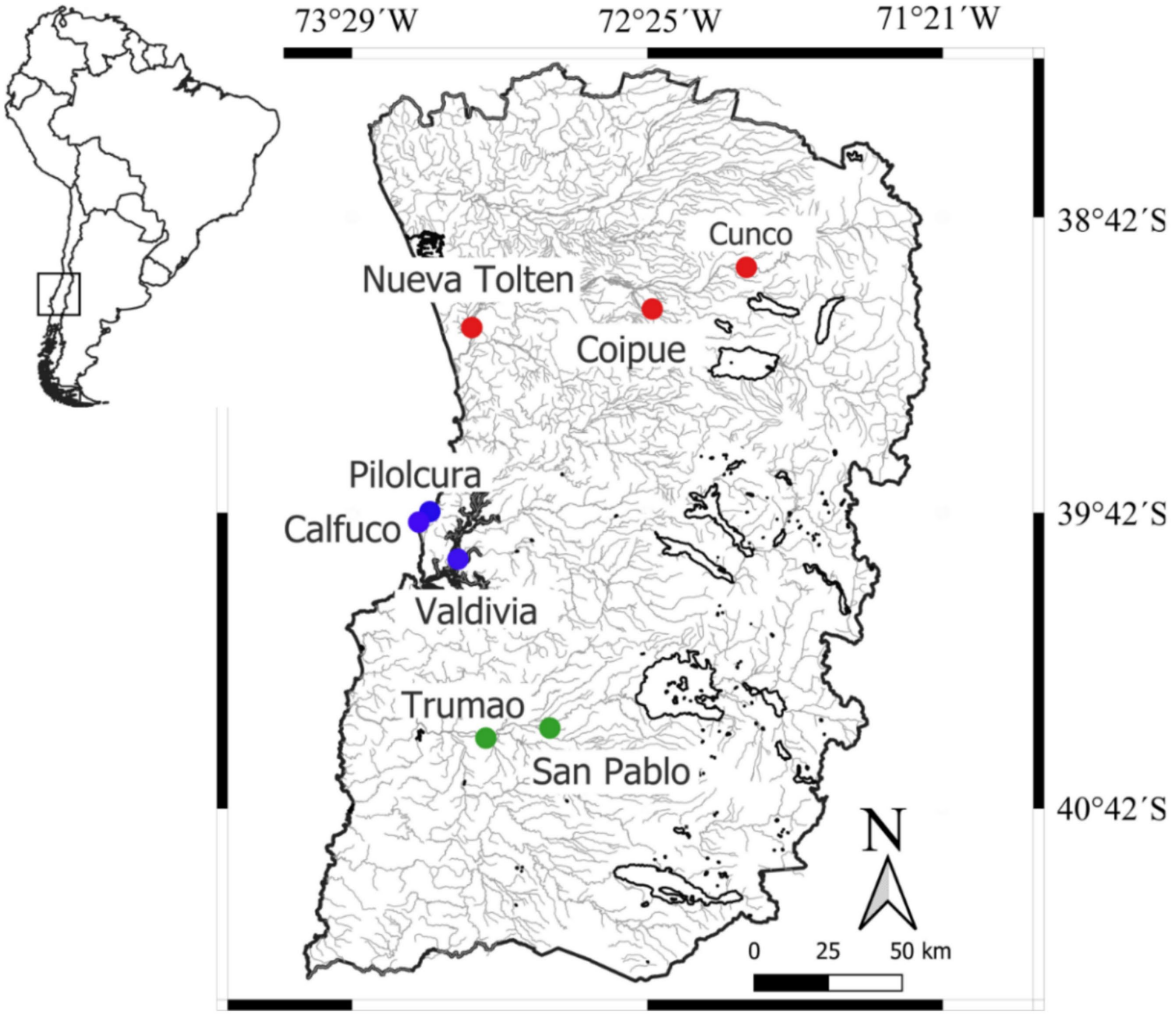
Location of the sampling sites within the temperate rainforest ecoregion of south-central Chile. The sampling sites in the Toltén River, Valdivia River and Río Bueno basins are indicated with red, blue and green circles, respectively.

Between January 2018 and January 2020, blood and mucous membrane samples were obtained from domestic dogs, invasive American minks, and native otters at three hydrographic basins within a 200 Km long study area, where inhabits both otters and the rural activities contribute to the presence of free-ranging dogs and minks (55, 56). Toltén River basin has remnant riparian forest coexisting with introduced commercial plantations of *Pinus* spp. and *Eucalyptus* spp. This area is located near the current northern distributional limit of the southern river otter and has previously been used as a study site to investigate trophic, population, and genetic of the species (57). In Cunco (upper basin) we sampled dogs and southern river otters, while in Coipue (mid-basin) we sampled dogs only and Nueva Toltén (lower basin) we sampled dogs, minks, and southern river otters (Figure 1). Southward, in the Valdivia River basin we sampled dogs and minks in the lower valley around Valdivia city. Furthermore, Calfuco and Pilolcura are two remote coastal localities characterized by a diverse rocky intertidal and subtidal community, which supports a stable population of marine otters (58, 59). Here we sampled only marine otters in Pilolcura and marine otters with minks in Calfuco (Figure 1). Finally, in Río Bueno basin (Figure 1) we sampled minks and dogs in San Pablo-Trumao (mid basin). Although the riparian forest in this area remains relatively undisturbed and provides important breeding and reproductive habitat for southern river otter (42, 60), no southern freshwater otter were captured.

Otters were lived captured using Victor® No 1.0 padded traps (Woodstream Corp., Lancaster, United States) to minimize the risk of injury. The sampled otters were previously analyzed for leptospirosis and toxoplasmosis (61). Minks were captured using single-door modify Tomahawk traps of 60 cm × 13 cm × 13 cm (Hazelhurst, United States) baited with canned fish or mink anal gland lures (62, 63). Both trap types were checked every 12 h (63–65). To minimize the likelihood of recapturing the same individual, sampling points were situated more than 10 km apart, exceeding the reported home range for these species (41, 66). Captured otters were anaesthetized with a combination of 5.3 mg/kg ketamine hydrochloride (Ketamil 111.56 mg/mL; Ilium Veterinary Products, Glendenning, Australia) and 26.5 μg/kg dexmedetomidine hydrochloride (Dexdomitor® 0.5 mg/mL; Zoetis, Parsippany, United States), administered via intramuscular injection (67, 68). Cardiorespiratory rate, oxygen saturation, body temperature, blood pressure, and depth of anesthesia through muscle relaxation and reflex responses were continuously monitored (67). Serological samples were obtained by venipuncture of the cephalic, jugular, or cranial vena cava to extract 6 mL of blood with anticoagulant-free tubes centrifuged *in situ* at 3000 rpm for 10 min within three hrs. of collection. In addition, conjunctival, tonsillar, and rectal mucosal swabs were obtained using sterile cotton-tipped applicators, which were placed into cryotubes containing viral transport medium and immediately frozen in liquid nitrogen (69). Thus, the samples were transported in liquid nitrogen to Health Ecosystem Laboratory, Universidad Andrés Bello (Santiago, Chile), where it was stored at −80 °C until processing.

Thirty minutes after the collection of serum and mucous samples, an intramuscular injection of 26.5 μg/kg atipamezole (Antisedan® 5.0 mg/mL; Zoetis, Parsippany, USA) was administered as an anesthetic reversal agent. During anesthesia and recovery, the captured individuals were housed in a dark, thermally controlled tubular containment cage until their release at the capture site, once full recovery had been achieved (70, 71). Captured mink were anaesthetized with a combination of 10 mg/kg ketamine hydrochloride and 25 μg/kg dexmedetomidine hydrochloride, and subsequently euthanized with an intracardiac injection of 5 mL sodium thiopental (67, 68). These actions are consistent with population control of invasive species programs based on capture/euthanasia and the application of trapping strategies specific to this species (62, 72). The samples from domestic dogs were collected with the consent of the owners, located no more than 10 km from the capture sites described above (41, 66). These samples were collected from brachiocephalic vein without anesthesia in unvaccinated individuals > 5 months old to avoid interference with maternal antibodies (73). The handling, capture, and sampling procedures for the animals analyzed were conducted in accordance with the protocols of Bioethics Committee of the Universidad Andrés Bello (Mustelids; Chile), Universidad Santo Tomás (dogs; Chile) and the Agencia Nacional de Investigación y Desarrollo de Chile (ANID), Fondecyt 1,171,417—Bioethics Approval No 007/2017. Additionally, the capture of otters was conducted under permit No. 1228 from the Subsecretaría de Pesca y Acuicultura de Chile.

### Serological and genetic analysis

A rapid ImmunoComb Canine test (BioGal Galed Laboratories Acs. Ltd., Kibbutz Galed, Israel) was applied to all blood serum samples following the manufacturer’s instructions. This commercial, ELISA-based immunoassay qualitatively detects immunoglobulin G (IgG) that reacts with the antigens of canine parvovirus and canine distemper virus. For the genetic detection of both diseases, the corresponding rectal, tonsillar, and conjunctival samples were separately subjected to DNA extraction and purification for the detection of canine parvovirus, and RNA extraction followed by reverse transcription to detect canine distemper (46, 74). DNA extraction was performed with the QIAamp DNA Mini kit (75), while RNA extraction was performed with the RNA-Solv Reagent kit (Omega Bio-Tek Inc., Norcross, United States) followed by the High-Capacity cDNA Reverse Transcription Kit (Applied Biosystems, Foster City, United States) for the reversal of RNA into cDNA (76). All extraction, purification, and reverse transcription procedures were carried out in accordance with the manufacturer’s instructions.

Polymerase chain reaction (PCR) was employed for the detection and cyclic amplification of a specific DNA sequence in a highly sensitive manner, enabling, among other applications, the diagnosis of infectious diseases by recognizing the genetic material of microorganisms in biological samples (47). Conventional PCR for parvovirus and distemper was performed by preparing a PCR 1X buffer containing 2 mM MgSO4, 0.1 mM dNTPs, 0.25 μM forward primer, 0.25 μM reverse primer, 1 U/25 μL Taq polymerase (M0267S, New England Biolabs, Inc., Ipswich, United States), and 1 μL template DNA, to a final volume of 25 μL per tube. CPV primers amplify an 83 bp fragment of the capsid VP2 protein, flanked by the forward primer 5´-ACAAGATAAAAGACGTGGTGTAACTCAA-3′ and the reverse primer 5´-CAACTTCAGCTGGTCTCATAATAGT-3′, while CDV primers detect and amplify a 388 bp fragment of the H protein gene, flanked by the forward primer 5’-TTTGGGGCAACACCTATGGATCAAGT-3′ and the reverse primer 5’-CTCCGGATGGCTTACCAT-3′ (46). For both sequences, the PCR program in the thermocycler consisted of an initial denaturation at 94° C for 5 min, followed by denaturation at 94° C for 30 s, annealing at 49° C for 30 s, elongation at 72° C for 30 s, elongation at 72° C for 10 min, and holding at 4° C for 15 min, all this repeated for 35 cycles. The Nobivac Puppy DP vaccine (Merck and Co., Inc., Rahway, United States), containing attenuated active strains of CPV (strain 154) and CDV (strain Onderstepoort), was used as a positive control, while molecular biology-grade distilled water was used as a negative control and nuclease free water as negative control. Subsequently, an electrophoretic run was performed on a 2% agarose gel in TAE 1X buffer at 100 volts for 30 min for qualitative DNA analysis, and for 40 min in the case of PCR products. A 100 bp DNA ladder was used as a molecular weight marker. The bands were stained with Gel and visualized under a trans illuminator Ultraviolet Viewer model UV1 (Extragene Inc., Taichung, Taiwan).

Quantitative real-time PCR with high-resolution melting analysis (q-PCR-HRM) was applied for parvovirus in order to differentiate amplicons based in their melting temperature (T_m_) to detects changes that can occur even in a single position of the sequence (48, 49, 77). HRM analysis establishes a high-resolution dissociation curve that differentiates DNA sequences based on their T_m_, defined as the temperature at which half of the DNA fragment is denatured or separated. This value depends on the conformation of the base pairs in the sequence. We used a mixture of the q-PCR Brilliant II SYBR Green qPCR Master Mix Kit 2X (Agilent Technologies, Santa Clara, USA), diluted to 1X, with the same primers used in conventional PCR at a concentration of 0.25 μM, and a reference dye at 30 nM. Added to this was 1 μL of DNA, with the final volume per tube adjusted to 20 μL using molecular biology-grade water. The q-PCR program was performed on an Agilent Technologies AriaMx and consisted of an initial denaturation at 94 °C for 5 min, followed by 40 cycles of denaturation at 94 °C for 30 s, annealing at 49 °C for 30 s, and both initial and final elongation at 72 °C for 30 s. The amplification reaction was monitored in the FAM channel for the samples and in the HEK channel for the reference dye. The peak T_m_ of the sample amplicons was compared with that of the positive control to verify whether there was molecular correspondence between the two types of sequences (77). The HRM procedure consisted of one cycle at 95 °C for 30 s, 65 °C for 30 s, and 95 °C for 30 s, with temperature increments of 0.2 °C from 65 °C to 95 °C. All serological and molecular analyses described were conducted in the Clinical Biochemistry Laboratory of the Medical Technology Department at Santo Tomás University, Osorno Campus, Chile.

For each species, the prevalence of parvovirus, distemper, and co-infection was defined as the proportion of seropositive individuals relative to the total number of animals captured. The uncertainty associated with prevalence was quantified using exact binomial 95% confidence interval calculated according to the Clopper–Pearson method (78). Potential statistical differences between age classes (juvenile, adult), sexes (female, male), and hydrographic basins were assessed using Fisher’s exact test (79). Both test were performed in the base package of R 4.1.1 (80).

## Results

A total of 81 individuals were sampled in the study area, from which blood serum and mucous membrane samples were collected. Of these, 86.4% (70 individuals) were invasive species, with *N. vison* and *C. lupus familiaris* representing 51.9% (n = 42) and 34.6% (n = 28) of the total sample, respectively. The remaining 11 individuals belonged to native mustelids, comprising eight marine otters and three southern river otters. All marine otters were captured in Valdivia River basin (4 in Calfuco and 4 in Pilolcura), where 62% of the mink individuals (n = 26) were also captured. In contrast, 50% of the dogs in the sample were obtained from the Toltén River basin (Table 1).

**Table 1:** Sample size (N) and serological and genetic prevalence of parvovirus and distemper (mean % and exact binomial 95% confidence intervals) for four species across three river basins in the temperate rainforest ecoregion of south-central Chile.

Hydrographic basin		Southern river otters	Marine otters	Minks	Dogs
Tolten River	N	3	0	8	14
	Seropositivity parvovirus	0 [0% (0–70.8%)]	-	0 [0% (0–36.9%)]	13 [92.9% (66.1–99.8%)]
	Seropositivity distemper	0 [0% (0–70.8%)]	-	0 [0% (0–36.9%)]	3 [21.4% (4.7–50.8%)]
	Seropositivity both diseases	0 [0% (0–70.8%)]	-	0 [0% (0–36.9%)]	3 [21.4% (4.7–50.8%)]
	Parvovirus (S+/PCR+)	0 [0% (0–70.8%)]	-	0 [0% (0–36.9%)]	4 [28.6% (0.1–58.1%)]
	Parvovirus (S-/PCR+)	2 [66.7% (9.4–99.2%)]	-	0 [0% (0–36.9%)]	0 [0% (0–23.1%)]
	Distemper (S-/PCR-)	0 [0% (0–70.8%)]	-	0 [0% (0–36.9%)]	4 [28.6% (0.1–58.1%)]
Valdivia River	N	0	8	26	9
	Seropositivity parvovirus	-	6 [75% (34.9–96.8%)]	1 [3.8% (0.01–38.5%)]	9 [100% (66.4–100%)]
	Seropositivity distemper	-	0 [0% (0–36.9%)]	0 [0% (0–13.2%)]	6 [66.7% (29.9–92.5%)]
	Seropositivity both diseases	-	0 [0% (0–36.9%)]	0 [0% (0–13.2%)]	6 [66.7% (29.9–92.5%)]
	Parvovirus (S+/PCR+)	-	2 [25% (3.2–65.1%)]	1 [3.8% (0.01–38.5%)]	6 [66.7% (29.9–92.5%)]
	Parvovirus (S+/PCR-)	-	4 [50% (15.7–84.3%)]	0 [0% (0–13.2%)]	3 [33.3% (7.5–70.1%)]
	Parvovirus (S-/PCR+)	-	0 [0% (0–36.9%)]	3 [11.5% (2.4–30.1%)]	0 [0% (0–33.6%)]
	Distemper (S-/PCR-)	-	0 [0% (0–36.9%)]	3 [11.5% (2.4–30.1%)]	0 [0% (0–33.6%)]
Río Bueno	N	0	0	8	5
	Seropositivity parvovirus	-	-	0 [0% (0–36.9%)]	3 [60% (14.7–94.7%)]
	Seropositivity distemper	-	-	0 [0% (0–36.9%)]	0 [0% (0–52.2%)]
	Seropositivity both diseases	-	-	0 [0% (0–36.9%)]	0 [0% (0–52.2%)]
	Parvovirus (S+/PCR+)	-	-	4 [50% (15.7–84.3%)]	0 [0% (0–52.2%)]
	Parvovirus (S-/PCR+)	-	-	0 [0% (0–36.9%)]	1 [20% (0.5–71.6%)]
	Distemper (S-/PCR-)	-	-	0 [0% (0–36.9%)]	0 [0% (0–52.2%)]

All serological samples from dogs in Valdivia River basin, 92.9% of dogs from Toltén River basin (13 of 14 individuals), and 60% of dogs from Río Bueno basin (3 of 5 individuals) tested seropositive for either parvovirus alone or parvovirus in combination with distemper. In addition, canine distemper was consistently detected together with parvovirus in 21.4% of the dogs from Toltén River basin (3 of 14 individuals) and 66.7% of the dogs from Valdivia River basin (6 of 9 individuals). Of the 26 minks captured at Valdivia River basin, only one (3.8%) tested seropositive for parvovirus, but 75% of marine otters (4 in Calfuco and 2 of 4 in Pilolcura) tested seropositive for parvovirus (Table 1). Furthermore, 66.6% of southern river otters tested from Toltén River basin (2 of 3 individuals) were positive for parvovirus by conventional PCR, although they were negative with serological analysis. In the sample from Valdivia River basin, all dogs, 1 of 26 minks, and 7 of 8 marine otters tested positive by both serological analysis and conventional PCR. In Río Bueno basin, one dog tested negative for both the serological reaction and positive for conventional PCR, while one mink was positive by serological and conventional PCR analysis (Table 1). All samples serologically positive for canine distemper were negative for conventional PCR. Intraspecifically, no significant differences were detected in the seroprevalence of either pathology with respect to age class (juvenile vs. adult), sex (female vs. male), or study site (Fisher’s exact test, *p* > 0.11). An exception was observed for parvovirus and distemper seropositivity, which differed significantly between dogs from the Valdivia River basin (66.7%) and those from the Río Bueno basin (0%; Fisher’s exact test, *p* = 0.031).

**Figure 2:**
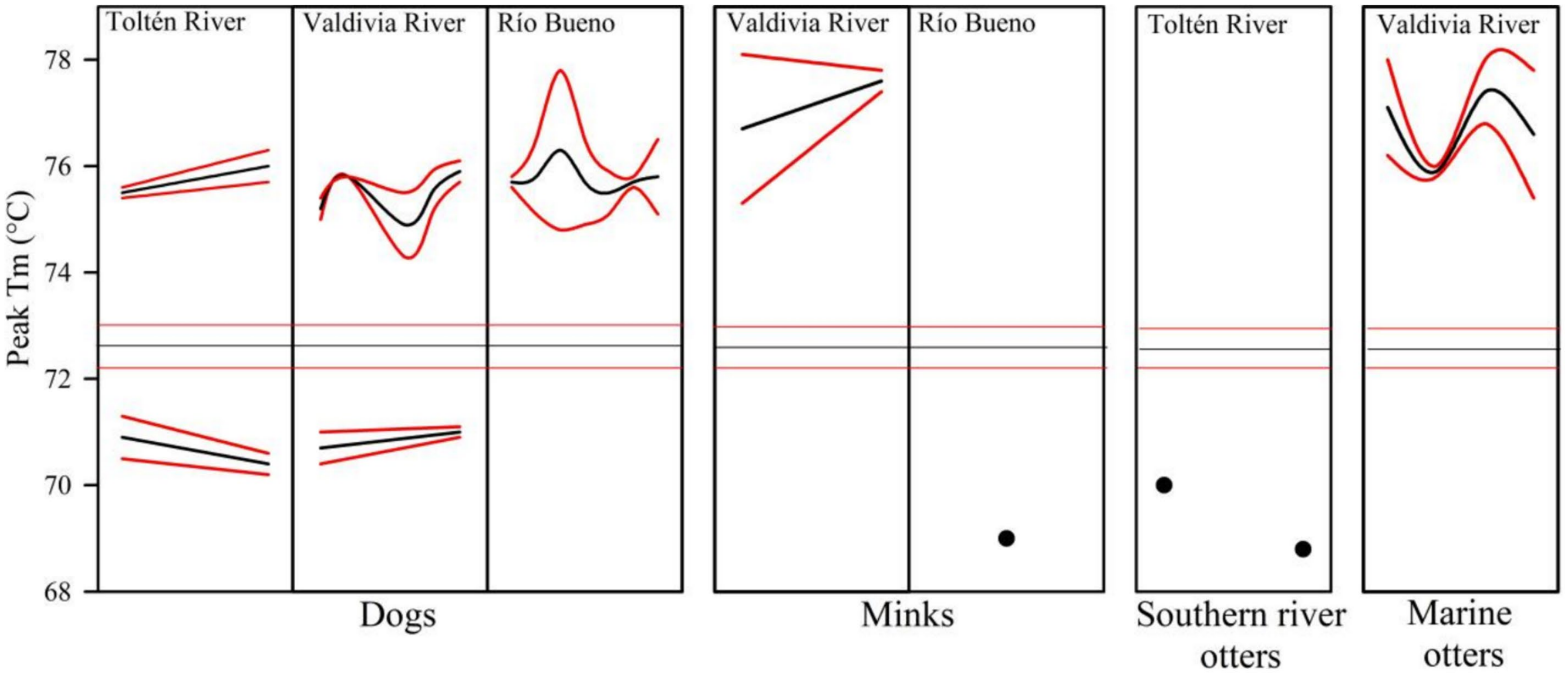
Melting temperature (T_m_) peaks obtained by qPCR-HRM for the VP2 gene of parvovirus in four species across three river basins. Mean (black lines) and 95% confidence intervals (red lines) are shown. Black dots denote individual T_m_ values, and horizontal lines indicate T_m_ values in the control kit.

In the qPCR-HRM analysis, all parvovirus-positive samples identified by conventional PCR exhibited melting temperatures (T_m_) that differed markedly from the T_m_ range of the positive control. Based on this pattern, two distinct genotypic profiles were identified for the nucleotide fragment analyzed in parvovirus. A group exhibiting higher T_m_ values than the control sample included dogs from Río Bueno basin, as well as minks and marine otters from Valdivia River basin. In contrast, parvovirus DNA fragments from minks in Río Bueno basin and southern river otters in Toltén River basin displayed lower T_m_ values than the control. Finally, both genotypic profiles were detected in dogs tested from Toltén River basin and Valdivia River basin (Figure 2).

## Discussion

Our findings support that the coexistence of domestic dogs and American mink facilitates the diversification of canine parvovirus among native aquatic mustelids in the temperate rainforest of south-central Chile. This pattern was evidenced not only by the presence of both structural variants of parvovirus in minks and dogs (detected through qPCR analysis of the DNA fragment and inferred from the T_m_ values), but also by the detection of genotypic variants between native otters from south-central Chile.

Between 2009 and 2016, both parvovirus and distemper were detected in southern river otters. During this period, the role of mink as a reservoir of these pathogens, and as a transmission bridge facilitated by its interactions with domestic dogs was described (25, 41). However, these findings contrasted with the absence of canine parvovirus reported in minks from samples collected between 2015 and 2016 within its current distribution in Chilean Patagonia (81). Both types of responses suggest that the infection and dispersion of parvovirus in minks was associated with population growth and expansion of its geographical distribution, which intensifies the intraspecific and community interactions and thereby increase the probability of infection by emerging diseases (81–84). In this sense, interactions between minks and dogs had likely favored the spread of canine parvovirus in both aquatic native otters from central- southern Chile. In addition, the high serological prevalence and low percentage of genetic detection of parvovirus in marine otters is indicative of its establishment in the population and the capacity of the hosts to become infected and recover (85–87).

The VP-2 gene fragment of parvovirus from marine otters captured in Valdivia River basin exhibited T_m_ values similar to dogs and minks from the same locality. Indeed, dogs registered all parvovirus sequences detected in our study in nearly all study sites, minks also had all sequences but with differences between study sites. These genotypic and study site correspondence suggests the ecological interactions between otters and minks in the transmission of parvovirus, and domestic dogs as the main reservoir. In the freshwater environments of this ecoregion, southern river otters latrines are landscape features that attract a host of species and create interspecific connectivity scenarios in which minks and southern river otters frequently co-occur, thereby constituting a potential source of pathogen transmission between native and invasive species (25, 42).

In the coastal environments of south-central Chile, frequent co-occurrence between native fauna and minks (and potentially with domestic dogs) is also expected to occur at marine otter latrine sites. However, by addressing the challenges associated with accessing the complex locations of their latrines and deploying camera traps within them, it will be possible to document their co-occurrence with other mammals, including rodents, minks, and dogs (19, 88–90). Furthermore, the fact that mink from Río Bueno basin carried the genotypic variant with the lowest T_m_ value, which was also detected in southern river otters from Toltén River basin, supports the notion that minks may function not only as a transmission bridge for canine parvovirus, but also as a reservoir of genotypic variants present in rural dogs (38, 91, 92). Therefore, as the exposure rate to parvovirus depends on the density and/or prevalence of infection in the invasive host, the most probable interpretation our results is a meta-reservoir created by invasive mink and domestic dogs associated with population densities and frequency of contact (41).

Within the Chilean context, minks are susceptible to infection and capable of transmitting multiple viral, bacterial, and parasitic diseases, some of which are zoonotic (61, 81, 93, 94). The synanthropy of minks, associated with attacks on farms and animal breeding centers (72, 95), would favor interaction with domestic dogs and rodents. In the temperate rainforest ecoregion of south-central Chile, 11 endemic rodents species have been described coexisting actively with three introduced rodent species (rats [*Rattus rattus, R. norvegicus*], and house mice [*Mus musculus*]). This interaction has likely generated a complex network of disease transmission, including hantavirus, toxoplasmosis, and rabies (42, 93). Additionally, since the home range of minks can exceed 5 km (96, 97), the same individuals may move between riverine and coastal areas, co-occurring with either species of native otters through the attractant effect of their latrines. Under this premise, biological control of mink in south-central Chile is recommended. For example, to intensify current capture and euthanasia efforts (62, 63), especially toward the northern limits of their current distribution to reduce their geographic dispersal rate. Also is advisable to incorporate serological and genetic analyses into these programs, in order to generate a geo-referenced database that enables the identification of spatio-temporal patterns in disease transmission (98, 99). Furthermore, regulatory measures concerning the implementation of vaccination schedules and territorial restrictions for rural domestic dogs, alongside controls on the access of minks to farms, could substantially reduce dog–mink co-occurrence. This, in turn, would lessen their pathological impact as both a transmission bridge and a key genetic reservoir for canine parvovirus, canine distemper, and other diseases linked to the coexistence of native and invasive species in south-central Chile.

## Data Availability

The original contributions presented in the study are included in the article/Supplementary material, further inquiries can be directed to the corresponding authors.

## References

[1] Daszak P Cunningham AA Hyatt AD. Emerging infectious diseases of wildlife—threats to biodiversity and human health. Science. (2000) 287:443–9. doi: 10.1126/science.287.5452.443, PMID: 10642539

[2] Salo P Korpimäki E Banks PB Nordström M Dickman CR. Alien predators are more dangerous than native predators to prey populations. Proc Biol Sci. (2007) 274:1237–43. doi: 10.1098/rspb.2006.0444, PMID: 17360286 PMC1950296

[3] Vitousek PM D’Antonio CM Loope LL Westbrooks R. Introduced species: a significant component of human-caused global change. N Z J Ecol. (1997) 21:1–16.

[4] Johnson PTJ de Roode JC Fenton A. Why infectious disease research needs community ecology. Science. (2015) 349:1259504. doi: 10.1126/science.1259504, PMID: 26339035 PMC4863701

[5] Young HS Parker IM Gilbert GS Guerra AS Nunn CL. Introduced species, disease ecology, and biodiversity–disease relationships. Trends Ecol Evol. (2017) 32:41–54. doi: 10.1016/j.tree.2016.09.008, PMID: 28029377

[6] Jones KE Patel NG Levy MA Storeygard A Balk D Gittleman JL . Global trends in emerging infectious diseases. Nature. (2008) 451:990–3. doi: 10.1038/nature06536, PMID: 18288193 PMC5960580

[7] Keele BF Van Heuverswyn F Li Y Bailes E Takehisa J Santiago ML . Chimpanzee reservoirs of pandemic and nonpandemic HIV-1. Science. (2006) 313:523–6. doi: 10.1126/science.1126531, PMID: 16728595 PMC2442710

[8] Plowright RK Reaser JK Locke H Woodley SJ Patz JA Becker DJ . Land use-induced spillover: a call to action to safeguard environmental, animal, and human health. Lancet Planet Health. (2021) 5:e237–45. doi: 10.1016/S2542-5196(21)00031-0, PMID: 33684341 PMC7935684

[9] Wolfe ND Dunavan CP Diamond J. Origins of major human infectious diseases. Nature. (2007) 447:279–83. doi: 10.1038/nature05775, PMID: 17507975 PMC7095142

[10] Zhou P Yang XL Wang XG Hu B Zhang L Zhang W . A pneumonia outbreak associated with a new coronavirus of probable bat origin. Nature. (2020) 579:270–3. doi: 10.1038/s41586-020-2012-7, PMID: 32015507 PMC7095418

[11] Armesto JJ León-Lobos P Kalin-Arroyo MT. Los bosques templados del sur de Chile y Argentina: Una isla biogeográfica In: Armesto JJ Villagrán C Kalin-Arroyo MT, editors. Ecología de los bosques nativos de Chile. Chile: Editorial Universitaria (1995). 23–8.

[12] Myers N Mittermeier RA Mittermeier CG da Fonseca GAB Kent J. Biodiversity hotspots for conservation priorities. Nature. (2000) 403:853–8. doi: 10.1038/35002501, PMID: 10706275

[13] Catchpole S Barría EM González PS Rivera R. Population and reproductive structure in the endangered and highly endemic freshwater crab *Aegla concepcionensis* (Decapoda: Pleocyemata: Aeglidae) from Chile. Acta Zool. (2023) 104:216–30. doi: 10.1111/azo.12408, PMID: 41131858

[14] Echeverría C Coomes D Salas J Rey-Benayas JM Lara A Newton AC. Rapid deforestation and fragmentation of Chilean temperate forests. Biol Conserv. (2006) 130:481–94. doi: 10.1016/j.biocon.2006.01.017

[15] Urrutia-Jalabert R González ME González-Reyes A Lara A Garreaud R. Climate variability and forest fires in central and south-Central Chile. Ecosphere. (2018) 9:e02171. doi: 10.1002/ecs2.2171

[16] Chehébar C. A survey of the southern river otter *Lutra provocax* Thomas in Nahuel Huapi national park. Argentina Biolog Conservation. (1985) 32:299–307.

[17] Medina-Vogel G González-Lagos C. Habitat use and diet of endangered southern river otters *Lontra provocax* in a predominantly palustrine wetland in Chile. Wildl Biol. (2008) 14:211–20. doi: 10.2981/0909-6396(2008)14[211:HUADOE]2.0.CO;2

[18] Medina-Vogel G Bartheld JL Álvarez- Pacheco R Delgado-Rodríguez C. Population assessment and habitat use by marine otter *Lontra felina* in southern Chile. Wildl Biol. (2006) 12:191–9. doi: 10.2981/0909-6396(2006)12[191:PAAHUB]2.0.CO;2

[19] Valqui J. The marine otter *Lontra felina* (Molina, 1782): a review of its present status and implications for future conservation. Mamm Biol. (2012) 77:75–83. doi: 10.1016/j.mambio.2011.08.004

[20] Fasola L Zucolillo P Roesler CI Cabello JL. A foreign carnivore: the case of American mink (*Neovison vison*) In: America S Jaksic EFM Castro SA, editors. Biological invasions in the south American Anthropocene: Global causes and local impacts. Switzerland AG: Springer Nature (2021). 255–99.

[21] Jaksic FM Iriarte JA Jiménez JE Martínez DR. Invaders without frontiers: cross-border invasions of exotic mammals. Biol Invasions. (2002) 4:157–73. doi: 10.1023/A:1020576709964

[22] Medina-Vogel G Barros M Organ JF Bonesi L. Coexistence between the southern river otter and the alien invasive north American mink in marine habitats of southern Chile. J Zool. (2013) 290:27–34. doi: 10.1111/jzo.12010

[23] Schüttler E Klenke R McGehee S Rozzi R Jax K. Vulnerability of ground-nesting waterbirds to predation by invasive American mink in the Cape Horn biosphere reserve, Chile. Biol Conserv. (2009) 142:1450–60. doi: 10.1016/j.biocon.2009.02.013

[24] Barrera R. Análisis de registros de ataques a fauna silvestre chilena por carnívoros domésticos: perro (*Canis lupus familiaris*) y gato (*Felis silvestris catus*) entre los años 2000 y 2016. Revista de Medicina Veterinaria e Investigación. (2018) 1:92–101. Available online at: https://www.researchgate.net/publication/325050121_Analisis_de_registros_de_ataques_a_fauna_silvestre_chilena_por_carnivoros_domesticos_perro_Canis_lupus_familiaris_y_gato_Felis_silvestris_catus_entre_los_anos_2000_y_2016

[25] Sepúlveda MA Singer RS Silva-Rodríguez EA Eguren A Stowhas P Pelican K. Invasive American mink: linking pathogen risk between domestic and endangered carnivores. EcoHealth. (2014) 11:409–19. doi: 10.1007/s10393-014-0917-z, PMID: 24604545

[26] Silva-Rodríguez EA Sieving KE. Influence of care of domestic carnivores on their predation on vertebrates. Conserv Biol. (2011) 25:808–15. doi: 10.1111/j.1523-1739.2011.01686.x, PMID: 21658128

[27] Silva-Rodríguez EA Sieving KE. Domestic dogs shape the landscape-scale distribution of a threatened forest ungulate. Biol Conserv. (2012) 150:103–10. doi: 10.1016/j.biocon.2012.03.008

[28] Vanak AT Gompper ME. Dogs (*Canis familiaris*) as carnivores: their role and function in intraguild competition. Mammal Rev. (2009) 39:265–83. doi: 10.1111/j.1365-2907.2009.00148.x

[29] Villatoro FJ Sepúlveda MA Stowhas P Silva-Rodríguez EA. Urban dogs in rural areas: human-mediated movement defines dog populations in southern Chile. Prev Vet Med. (2016) 135:59–66. doi: 10.1016/j.prevetmed.2016.11.004, PMID: 27931930

[30] Young JK Olson KA Reading RP Amgalanbaatar S Berger J. Is wildlife going to the dogs? Impacts of feral and free-roaming dogs on wildlife populations. Bioscience. (2011) 61:125–32. doi: 10.1525/bio.2011.61.2.7

[31] González-Acuña D Ortega-Vásquez R Rivera-Ramírez P Cabello-Cabalin J. Verdacht auf Staupe beim Graufuchs (*Pseudalopex griseus*) im mittleren Chile (Fallbericht). Z Jagdwiss. (2003) 49:323–6. doi: 10.1007/BF02189641, PMID: 41134348

[32] Kelman M Harriot L Carai M Kwan E Ward MP Barrs VR. Phylogenetic and geospatial evidence of canine parvovirus transmission between wild dogs and domestic dogs at the urban fringe in Australia. Viruses. (2020) 12:663. doi: 10.3390/v12060663, PMID: 32575609 PMC7354627

[33] Quintero-Gil C Rendon-Marín S Martínez-Gutierrez M Ruiz-Saenz J. Origin of canine distemper virus: consolidating evidence to understand potential zoonoses. Front Microbiol. (2019) 10:1982. doi: 10.3389/fmicb.2019.01982, PMID: 31555226 PMC6722215

[34] Vieira FV Hoffmann DJ Fabri CUF Bresciani KDS Gameiro R Flores EF . Circulation of canine parvovirus among dogs living in human–wildlife interface in the Atlantic Forest biome, Brazil. Helyon. (2018) 3:e00491. doi: 10.1016/j.helyon.2017.e00491 PMC5772843 29387822

[35] Wilkes RP. Canine distemper virus in endangered species: species jump, clinical variations, and vaccination. Pathogens. (2023) 12:57. doi: 10.3390/pathogens12010057, PMID: 36678405 PMC9862170

[36] Courtenay O Quinnell RJ Chalmers WSK. Contact rates between wild and domestic canids: no evidence of parvovirus or canine distemper virus in crab-eating foxes. Vet Microbiol. (2001) 81:9–19. doi: 10.1016/S0378-1135(01)00326-1, PMID: 11356314

[37] Miranda C Thompson G. Canine parvovirus: the worldwide occurrence of antigenic variants. J Gen Virol. (2016) 97:2043–57. doi: 10.1099/jgv.0.000540, PMID: 27389721

[38] Steinel A Parrish CR Bloom ME Truyen U. Parvovirus infections in wild carnivores. J Wildl Dis. (2001) 37:594–607. doi: 10.7589/0090-3558-37.3.594, PMID: 11504234

[39] Acosta-Jamett G Surot D Cortés M Marambio V Valenzuela C Vallverdu A . Epidemiology of canine distemper and canine parvovirus in domestic dogs in urban and rural areas of the Araucanía region in Chile. Vet Microbiol. (2015) 178:260–4. doi: 10.1016/j.vetmic.2015.05.012, PMID: 26013417

[40] Acosta-Jamett G Cunningham AA Cleaveland S. Serosurvey of canine distemper virus and canine parvovirus in wild canids and domestic dogs at the rural interface in the Coquimbo region, Chile. Eur J Wildl Res. (2015) 61:329–32. doi: 10.1007/s10344-014-0886-0

[41] Barros M Pons DJ Moreno A Vianna J Ramos B Dueñas F . Domestic dog and alien north American mink as reservoirs of infectious diseases in the endangered southern river otter. Austral J Vet Sci. (2022) 54:65–75. doi: 10.4067/S0719-81322022000200065

[42] Santibañez A. Invasión de especies y sus implicancias para la salud de mamíferos endémicos del bosque templado-lluvioso del centro-sur de Chile. PhD Thesis,. Chile: Universidad Santo Tomás (2025).

[43] Sepúlveda MA Singer RA Silva-Rodríguez EA Stowhas P Pelican K. Domestic dogs in rural communities around protected areas: conservation problem or conflict solution? PLoS One. (2014) 9:e86152. doi: 10.1371/journal.pone.0086152, PMID: 24465930 PMC3896434

[44] Bergmann M Freisl M Hartmann K Speck S Truyen U Zablotski Y . Antibody response to canine parvovirus vaccination in dogs with hypothyroidism treated with levothyroxine. Vaccine. (2021) 9:180. doi: 10.3390/vaccines9020180, PMID: 33672564 PMC7924029

[45] Harmening DM. Modern blood banking and transfusion practices. US: F.A. Davis Co (2019). 669 p.

[46] Decaro N Desario C Elia G Martella V Mari V Lavazza A . Evidence for immunisation failure in vaccinated adult dogs infected with canine parvovirus type 2c. New Microbiol. (2008) 31:125–30. Available online at: https://www.researchgate.net/publication/5414798_Evidence_for_immunisation_failure_in_vaccinated_adult_dogs_infected_with_canine_parvovirus_type_2c PMID: 18437851

[47] Yang S Rothman RE. PCR-based diagnostics for infectious diseases: uses, limitations and future applications. Lancet Infect Dis. (2004) 4:337–48. doi: 10.1016/S1473-3099(04)01044-8, PMID: 15172342 PMC7106425

[48] Erali M Voelkerding KV Wittwer CT. High-resolution melting applications for clinical laboratory medicine. Exp Mol Pathol. (2008) 85:50–8. doi: 10.1016/j.yexmp.2008.03.012, PMID: 18502416 PMC2606052

[49] Hinsberger A Saint-Germain ST Guerrero P Blachère-López C López-Ferber M Bayle S. A combination of real-time PCR and high-resolution melting analysis to detect and identify CpGV genotypes involved in type I resistance. Viruses. (2019) 11:723. doi: 10.3390/v11080723, PMID: 31390849 PMC6723291

[50] Lombal AJ Wenner TJ Burridge CP. Assessment of high-resolution melting (HRM) profiles as predictors of microsatellite variation: an example in Providence petrel (Pterodroma solandri). Genes Genomics. (2015) 37:977–83. doi: 10.1007/s13258-015-0327-9

[51] Wittwer CT Reed GH Gundry CN Vandersteen JG Pryor RJ. High-resolution genotyping by amplicon melting analysis using LCGreen. Clin Chem. (2003) 49:853–60. doi: 10.1373/49.6.853, PMID: 12765979

[52] Bingga G Liu Z Zhang J Zhu Y Lin L Ding S . High resolution melting curve analysis as a new tool for rapid identification of canine parvovirus type 2 strains. Mol Cell Probes. (2014) 28:271–8. doi: 10.1016/j.mcp.2014.08.001, PMID: 25159576

[53] Peterlana D Puccetti A Corrocher R Lunardi C. Serological and molecular detection on human *parvovirus B19* infection. Clin Chim Acta. (2006) 372:14–23. doi: 10.1016/j.ccc.2006.04.018 16765338

[54] Sun Y Cheng Y Lin P Zhang H Yi L Tong M . Simultaneous detection and differentiation of canine parvovirus and feline parvovirus by high resolution melting analysis. BMC Vet Res. (2019) 15:141. doi: 10.1186/s12917-019-1898-5, PMID: 31077252 PMC6511188

[55] Gálvez N Infante J Fernández A Díaz J Petracca L. Land use intensification coupled with free-roaming dogs as potential defaunation drivers of mesocarnivores in agricultural landscapes. J Appl Ecol. (2021) 58:2962–74. doi: 10.1111/1365-2664.14026

[56] Schüttler E Rozzi R Jax K. Towards a societal discourse on invasive species management: a case study of public perceptions of mink and beavers in Cape Horn. J Nat Conserv. (2011) 19:175–84. doi: 10.1016/j.jnc.2010.12.001

[57] Medina-Vogel G. Barros-Lama M. Klarian-Klarian A. Calvo-Mac C. (2021) Distribución, abundancia y riesgos para la conservación del huillín (*Lontra provocax*) en la cuenca del río Allipén y Toltén. Informe final Proyecto FIPA 2018–28. Santiago of Chile. Fondo de Investigación Pesquera y de Acuicultura.

[58] Córdova O Rau JR Suazo CG Arriagada A. Comparative study of the feeding ecology of the top predator *Lontra felina* (Molina, 1782) (Carnivora: Mustelidae) in Chile. Rev Biol Mar Oceanogr. (2009) 44:429–38. doi: 10.4067/S0718-19572009000200016, PMID: 27315006

[59] Medina-Vogel G Delgado-Rodríguez C Álvarez R Bartheld JL. Feeding ecology of the marine otter (*Lutra felina*) in a rocky seashore of the south of Chile. Mar Mamm Sci. (2006) 20:134–44. doi: 10.1111/j.1748-7692.2004.tb01144.x, PMID: 41131858

[60] Santibañez A Barría EM Barros M Coccia C Medina-Vogel G. First detection of *Lontra provocax* in an unexplored hydrological basin of Central-Southern Chile. Aquat Mamm. (2024) 50:13–8. doi: 10.1578/AM.50.1.2024.13

[61] Calvo-Mac C Barros-Lama M Martínez-Leiva GK Salgado M Medina-Vogel G. Exposure to pathogenic *Leptospir*a and *toxoplasma gondii* in endangered native otters of the Valdivian temperate rainforest ecoregion in Chile. Aquat Mamm. (2024) 50:39–44. doi: 10.1578/AM.50.1.2024.39

[62] Medina-Vogel G Barros M Monsalve R Pons DJ. Assessment of the efficiency in trapping north American mink (*Neovison vison*) for population control in Patagonia. Rev Chil Hist Nat. (2015) 88:5174. doi: 10.1186/s40693-015-0040-8, PMID: 41136398

[63] Medina-Vogel G Muñoz F Moeggenberg M Calvo-Mac C Barros-Lama M Ulloa N . Improving trapping efficiency for control of American mink (*Neovison vison*) in Patagonia. Animals. (2022) 12:142. doi: 10.3390/ani12020142, PMID: 35049765 PMC8772562

[64] Blundell GM Kern JW Bowyer R Duffy LK. Capturing river otters: a comparison of Hancock and leg-hold traps. Wildlife Society Bulletin (1973–2006). (1999) 27:184–92.

[65] Schiaffini MI Beckles AA Guisasola M Bauer GG. Manejo del visón americano *Neogale vison* (Carnivora: Mustelidae) en el Parque Nacional Los Alerces, República Argentina. Notas Mamiferos Sudam. (2022) 4:001–10. doi: 10.31687/SaremNMS22.6.3

[66] Barros M Cabezón O Dubey JP Almería S Ribas MP Escobar LE . *Toxoplasma gondii* infection in wild mustelids and cats across an urban–rural gradient. PLoS One. (2018) 13:e0199085. doi: 10.1371/journal.pone.0199085, PMID: 29924844 PMC6010287

[67] Barros-Lama M Azat C Tardone R Medina-Vogel G. Chemical immobilisation of the wild Patagonian otter (*Lontra provocax*) and the north American mink (*Neovison vison*). Austral J Vet Sci. (2021) 53:127–31. doi: 10.4067/S0719-81322021000200127

[68] Soto-Azat C Boher F Flores G Mora E Santibañez A Medina-Vogel G. Reversible anesthesia in wild marine otters (*Lontra felina*) using ketamine and medetomidine. J Zoo Wildl Med. (2006) 37:535–8. doi: 10.1638/05-110.1, PMID: 17315440

[69] Druce J Garcia K Tran T Papadakis G Birch C. Evaluation of swabs, transport media, and specimen transport conditions for optimal detection of viruses by PCR. J Clin Microbiol. (2012) 50:1064–5. doi: 10.1128/JCM.06551-11, PMID: 22205810 PMC3295134

[70] Creighton CM. Monitoring Equipment In: Bressan N Creighton CM, editors. An Introdiction to veterinary medicine engineering. Cham: Springer (2023). 13–26.

[71] Granholm M McKusick BC Westerholm FC Aspegren JC. Evaluation of the clinical efficacy and safety of intramuscular and intravenous doses of dexmedetomidine and medetomidine in dogs and their reversal with atipamezole. Vet Rec. (2007) 160:891–7. doi: 10.1136/vr.160.26.891, PMID: 17602104

[72] López R Clapperton BK Medina-Vogel G. A global review of the American mink *(Neovison vison)* removal techniques – Patagonia as a case study for their potential application. Gayana. (2023) 87:43–62. doi: 10.4067/S0717-65382023000100043, PMID: 27315006

[73] Chappuis G. Neonatal immunity and immunisation in early age: lessons from veterinary medicine. Vaccine. (1998) 16:1468–72. 9711790 10.1016/S0264-410X(98)00110-8 PMC7130764

[74] Elia G Decaro N Martella V Cirone F Lucente MS Lorusso E . Detection of canine distemper virus in dogs by real-time RT-PCR. J Virol Methods. (2006) 136:171–6. doi: 10.1016/j.jviromet.2006.05.004, PMID: 16750863

[75] QIAGEN. (2016). QIAamp DNA Mini and Blood Mini Handbook (4ª ed.). QIAGEN. Available online at: https://www.qiagen.com/us/resources/resourcedetail?id=62a200d6-faf4-469b-b50f-2b59cf738962andlang=en (Accessed January, 2025).

[76] Green MR Sambrook J. Amplification of cDNA generated by reverse transcription of mRNA: two-step reverse transcription-polymerase chain reaction (RT-PCR). Cold Spring Harb Protoc. (2019) 2019:190. doi: 10.1101/pdb.prot095190, PMID: 31043555

[77] Tong SYC Giffard PM. Microbiological applications of high-resolution melting analysis. J Clin Microbiol. (2012) 50:3418–21. doi: 10.1128/jcm.01709-12, PMID: 22875887 PMC3486245

[78] Zar JH. Biostatistical analysis. 5th ed. Upper Saddle River, NJ: Pearson Prentice Hall (2010).

[79] Sokal RR Rohlf FJ. Biometry: the principles and practice of statistics in biological research. 4th ed. New York: W. H. Freeman and Co. (2012).

[80] R Core Team. (2021). R: A language and environment for statistical computing (version 4.1.1) [computer software]. Available online at: R Foundation for Statistical Computing. https://www.R-project.org/ (Accessed August, 2025).

[81] Zapararte MB Ramírez-Pizarro F Landaeta-Aqueveque C Poulin E Ortega R Napolitano C. Molecular survey of parvoviruses and *Mycoplasma spp*. in invasive American mink (*Neovison vison*) from southern Chile. J Wildl Dis. (2021) 57:234–7. doi: 10.7589/WD-D-20-00047, PMID: 33635978

[82] Carrara F Giometto A Seymour M Rinaldo A Altermatt F. Inferring species interactions in ecological communities: a comparison of methods at different levels of complexity. Methods Ecol Evol. (2015) 6:895–906. doi: 10.1111/2041-210X.12363

[83] Medina-Vogel G Soto-Ampuero C Delgado-Parada N Molina-Maldonado G Calvo-Mac C. First video evidence of interaction between introduced American mink (*Neogale vison*) and endangered native Southern River otter (*Lontra provocax*). IUCN Otter Spec Group Bull. (2025) 42:179–85. Available online at: https://www.iucnosgbull.org/Volume42/Medina_Vogel_et_al_2025.pdf

[84] Rodríguez-Pastor R Escudero R Vidal D Mougeot F Arroyo B Lambin X . Density-dependent prevalence of *Francisella tularensis* in fluctuating vole populations, northwestern Spain. Emerg Infectious Dis. (2017) 23:1377–9. doi: 10.3201/eid2308.161194, PMID: 28726608 PMC5547778

[85] Gilbert AM Fooks AR Hayman DTS Horton DL Müller T Plowright R . Deciphering serology to understand the ecology of infectious diseases in wildlife. EcoHealth. (2013) 10:298–313. doi: 10.1007/s10393-013-0856-0, PMID: 23918033

[86] Ryser-Degiorgis MP. Wildlife health investigations: needs, challenges and recommendations. Vet Res. (2013) 9:223. doi: 10.1186/1746-6148-9-223, PMID: 24188616 PMC4228302

[87] Smith KF Acevedo-Whitehouse K Pedersen AB. The role of infectious diseases in biological conservation. Anim Conserv. (2009) 12:1–12. doi: 10.1111/j.1469-1795.2008.00228.x.

[88] Ebensperger LA Castilla JC. Selección de hábitat en tierra por la nutria marina, *Lutra felina*, en Isla Pan de Azúcar, Chile. Rev Chil Hist Nat. (1992) 65:429–34.

[89] Medina-Vogel G Calvo-Mac C Delgado-Parada N Molina-Maldonado G. The natural history of marine otter (*Lontra felina*) In: Ayala L Sánchez-Scaglioni R Medina-Vogel G, editors. Marine otters conservation. Cham: Springer (2024). 17–41.

[90] Ostfeld RS Ebensperger L Klosterman LL Castilla JC. Foraging, activity budget, and social behavior of the south American marine otter *Lutra felina* (Molina 1782). Natl Geogr Res. (1989) 5:422–38.

[91] Biek R Real LA. The landscape genetics of infectious disease emergence and spread. Mol Ecol. (2010) 19:3515–31. doi: 10.1111/j.1365-294X.2010.04679.x, PMID: 20618897 PMC3060346

[92] Siembieda JL Kock RA McCracken TA Newman SH. The role of wildlife in transboundary animal diseases. Anim Health Res Rev. (2011) 12:95–111. doi: 10.1017/S1466252311000041, PMID: 21615975

[93] Llanos-Soto S González-Acuña D. Knowledge about bacterial and viral pathogens present in wild mammals in Chile: a systematic review. Rev Chilena Infectol. (2019) 36:195–218. doi: 10.4067/S0716-10182019000200195, PMID: 31344156

[94] Ramírez-Pizarro F Silva-dela Fuente C Hernández-Orellana C López J Madrid V Fernández I . Zoonotic pathogens in the American mink in its southernmost distribution. Vector-Borne and Zoonotic Dis. (2019) 19:908–14. doi: 10.1089/vbz.2019.2445, PMID: 31390318

[95] Vehanen T Huusko A Bergman E Enefalk Å Louhi P Sutela T. American mink *(Neovison vison)* preying on hatchery and wild brown trout (*Salmo trutta*) juveniles in semi-natural streams. Freshw Biol. (2022) 67:433–44. doi: 10.1111/fwb.13852

[96] Bonesi L Macdonald DW. Differential habitat use promotes sustainable coexistence between the specialist otter and the generalist mink. Oikos. (2004) 106:509–19. doi: 10.1111/j.0030-1299.2004.13034.x

[97] Gerell R. Home ranges and movements of the mink *Mustela vison* Shreber in southern Sweden. Oikos. (1970) 21:160–73.

[98] Blanchong JA Robinson SJ Samuel MD Foster JT. Application of genetics and genomics to wildlife epidemiology. J Wildl Manag. (2016) 80:593–608. doi: 10.1002/jwmg.1064

[99] Storfer A Epstein B Jones M Micheletti S Spear SF Lachish S . Landscape genetics of the Tasmanian devil: implications for spread of an infectious cancer. Conserv Genet. (2017) 18:1287–97. doi: 10.1007/s10592-017-0980-4

